# Eucalyptus Oil-Induced Seizures in Children: A Single-Center Prospective Study

**DOI:** 10.7759/cureus.14109

**Published:** 2021-03-25

**Authors:** Dudipala Sai Chandar, Mandapuram Prashanthi, Chenalla Laxman Kumar, Chennadi Amith Kumar

**Affiliations:** 1 Pediatric Neurology, Prathima Institute of Medical Sciences, Karimnagar, IND; 2 Pediatrics, Prathima Institute of Medical Sciences, Karimnagar, IND

**Keywords:** eucalyptus oil, first seizures, ingestion, electroencephalography, neuroimaging

## Abstract

Objective

Eucalyptus oil (EO) is known to have proconvulsant properties. EO is present in many over-the-counter preparations and used orally or topically to treat many ailments. In this study, we seek to describe clinical features, neuroimaging, and electroencephalographic findings and follow up outcome in children with eucalyptus oil-induced seizures (EOIS).

Materials and methods

This was a single-center prospective observational study, conducted at a tertiary care hospital from South India over a period of two years. Children (up to 18 years of age) with a first afebrile seizure or breakthrough seizures with preexisting epilepsy and with a recent exposure to EO were included. Data from all the children including demographic data, exposure to EO, mode of exposure, time to onset of seizures in relation to exposure, duration of seizure, type of seizure, investigations, and antiepileptic drug therapy were noted. All the patients were followed up for recurrence of seizures for six to nine months.

Results

During the study period of two years, a total of 17 children met the inclusion criteria, with a median (range) age of 3.75 years (23 - 150 months) and 10 (59%) were male. Only one patient had breakthrough seizures and all other 16 children had a first episode of seizure. All the children had generalized tonic-clonic seizures with median duration of three minutes (30 sec - 30 minutes). The median (range) interval between EO exposure and the onset of seizures was 20 minutes (10 - 120 minutes). All the children had taken EO drops orally mixed in either water or milk in different amounts. All the patients' brain neuroimaging was normal. All the patients had normal electroencephalography except for four children. Only five patients were treated with antiepileptic drugs for two weeks and one patient with preexisting epilepsy was continued on the same antiepileptic drug. None of the patients had a recurrence of seizures during the follow-up period of six to nine months.

Conclusion

EOIS is an underrecognized and rare entity of seizures in children. EOIS can occur as first seizures or as breakthrough seizures in preexisting epilepsy patients. Despite the previous case reports in the literature quoting the seizurogenic potential of EO, this awareness is lacking in both clinicians and parents. We also recommend clinicians inquire about exposure to EO while approaching a child with first afebrile seizures or breakthrough seizures, which can prevent unnecessary investigations and long-term antiepileptic drug therapy.

## Introduction

In India, it is common practice to treat minor illnesses with essential oils like eucalyptus oil (EO) since ancient times. EO is a distilled volatile oil extracted from the leaves of the Eucalyptus tree. It is easily available over the counter (OTC) as cough drops, ointments, mouthwashes, and balms. It can be used orally, intranasally, or topically [1]. It is considered safe; however, it has adverse effects, with the most serious being neurological manifestations like encephalopathy, seizures, and ataxia. These manifestations can occur with overdose or therapeutic dosage [2]. EO has been identified to have seizurogenic properties. This seizurogenic effect of EO is due to 1, 8-cineole, which also known as eucalyptol. In the literature, some case series and individual case reports of eucalyptus oil-induced seizures (EOIS) have been reported, but pediatric case series are very few [3-9]. In children, the first episode of seizures or breakthrough seizures can occur with EO. But still, this is considered as an underrecognized or misdiagnosed entity, resulting in unnecessary hospitalization and investigations. Therefore, in this study, we attempt to describe clinical features, neuroimaging and electroencephalographic findings, and follow up outcomes in children with EOIS.

## Materials and methods

This study was a prospective observational study conducted at a tertiary hospital in South India for a period of two years (from March 2019 to February 2021). Children (up to 18 years of age) with first afebrile seizures or breakthrough seizures with preexisting epilepsy, with a recent exposure of EO, were included. Children with confirmed acute symptomatic seizures (due to fever, hypoglycemia, electrolyte disturbances, and systemic infections) and those with remote symptomatic etiology were excluded.

This study was approved by the institutional ethical committee and written informed consent was obtained from parents of enrolled children. Data for all the patients such as age, sex, past and family history of seizures and/or epilepsy was noted. The details of dose, route of exposure, the time between exposure and seizure onset, type, and duration of seizures were noted. The necessary investigations like blood glucose, serum electrolytes, electroencephalography (EEG), and neuroimaging were done, with the extent of evaluation varies on a case-to-case basis. All the children were followed up, physically or telephonically for a period of six to nine months. The data were presented as numbers and percentages.

## Results

During the study period of two years, a total of 17 children met the inclusion criteria, with a median (range) age of 3.75 years (23 - 150 months) and 10 (59%) were less than five years of age. Among these 17, 10 (59%) were male and seven (41%) were female (Table 1).

**Table 1:** Clinical characteristics of study subjects GTCS - generalized tonic-clonic seizures, EO - eucalyptus oil

No	Age(mo)/ sex	Amount taken	Route of exposure	Seizure onset (min)	Seizure type	Seizure duration (min)	Hospitalization	Indication for EO exposure
1	80/M	8 drops	Oral	30	GTCS	2	No	Cold
2	51/M	10 drops	Oral	20	GTCS	3	No	Cold
3	45/M	5 drops	Oral	15	GTCS	4	2	Cold
4	32/M	1mL	Oral	20	GTCS	8	1	Cold
5	82/F	10 drops	Oral	15	GTCS	2	No	Cold
6	40/M	4 mL	Oral	15	GTCS	25	2	Accidental
7	30/F	1 mL	Oral	120	GTCS	20	1	Pain abdomen
8	23/F	10drops	Oral	40	GTCS	1	1	Cold
9	80/M	1 mL	Oral	30	GTCS	2	2	Cold
10	150/M	2 mL	Oral + Local	30	GTCS	5	2	Toothache
11	102/M	2 mL	Oral	65	GTCS	3	No	Headache
12	33/F	5 drops	Oral	25	GTCS	4	No	Digestion
13	24/F	2 mL	Oral	20	GTCS	0.5	No	Cold
14	35/F	0.5 mL	Oral	15	GTCS	2	No	Cold
15	79/M	3 mL	Oral	10	GTCS	3	No	Cold
16	31/M	0.5mL	Oral	15	GTCS	5	No	Cold
17	66/F	5 mL	Oral +Local	10	GTCS	30	2	Sinusitis

Furthermore, 16 (94%) children had a first episode of seizures and only one child had breakthrough seizures. All the children had orally taken EO drops mixed either in water or milk in different amounts. Two children used EO as a local application along with oral ingestion. Only one child, who had a preexisting diagnosis of epilepsy and on regular antiepileptic drugs (AED), had accidental ingestion of 4 mL of EO. The quantity of liquid preparation of EO used was 5-10 drops (six of 17 children) and 0.5-5 mL (11 of 17 children). Common cold (n=11, 65%) was the most common cause for EO intake. None of these children had a past history of seizures except one child. All the children were developmentally normal and no one had a family history of seizures or epilepsy. Only three children had a history of exposure to EO in the past; these children now took an increased amount of EO.

In this study group, all the children developed generalized tonic-clonic seizures (GTCS), with median (range) seizure duration of three minutes (30 seconds - 30 minutes), and 13 (76%) children had seizure duration of less than five minutes. The median (range) interval between exposure and the onset of seizures was 20 minutes (10 - 120 minutes). Fourteen (82%) children developed seizures within 30 minutes after EO exposure. Of 17 children, eight (47%) were hospitalized. All the children were discharged within two days of admission. Investigations like blood glucose, serum electrolytes, calcium, and magnesium were done in all patients to rule out other causes of acute symptomatic seizures and were normal.

EEG was done in all the children and was normal in all except for four children. Three patients had generalized slowing and one patient had generalized spike and wave discharges. Ten children (59%) underwent neuroimaging (computerized tomography [CT] scan, five; magnetic resonance imaging [MRI], five) of the brain, which was normal in all (Table 2).

**Table 2:** Neuroimaging, EEG, and treatment profile of children with eucalyptus oil-induced seizures (EOIS) MRI - Magnetic resonance imaging, CT - Computerized tomography, EEG - Electroencephalography, AED - Antiepiletic drug, s&w - spike and wave discharges

No	Neuroimaging	EEG	AED used	Duration
1	MRI brain- Normal	Normal	No	No
2	Not done	Normal	No	No
3	MRI brain- Normal	Normal	No	No
4	MRI brain- Normal	Generalized slowing	Levetiracetam	2 weeks
5	CT brain- Normal	Normal	Valproate	1 week
6	Not done	Generalized s & w	Levetiracetam	Continuous
7	CT brain - Normal	Generalized s & w	Levetiracetam	2 weeks
8	Not done	Normal	No	No
9	Not done	Normal	No	No
10	MRI brain – Normal	Generalized slowing	Levetiracetam	2 weeks
11	CT brain – Normal	Normal	No	No
12	CT brain – Normal	Normal	No	No
13	Not done	Normal	No	No
14	CT brain – Normal	Normal	No	No
15	Not done	Normal	No	No
16	Not done	Normal	No	No
17	MRI brain - Normal	Generalized slowing	Levetiracetam	2 weeks

Five patients were treated with an AED (levetiracetam, four; valproate, one). The AEDs were tapered and stopped over a period of two weeks. AEDs were continued in a preexisting epilepsy patient. All the children were followed up for six to nine months and none had a recurrence of seizures. Patients were advised not to use EO again in any route.

## Discussion

EO is an aromatic, distilled volatile oil derived from the leaf of Eucalyptus, which belongs to the family Myrtaceae. Most EO production globally comes from *Eucalyptus globulus* [10]. EO has been known to have proconvulsant properties for centuries [11,12]. However, EOIS as an entity has not been recognized by clinicians due to lack of awareness of the proconvulsant effect of EO. With the increase in OTC availability and use of this product, EOIS is increasingly being diagnosed. There are several case series and reports of seizures provoked by EO in the literature [3-9,13-16].

EO is used widely across the world to treat ailments like the common cold, sinusitis, toothache, pain abdomen, headache, and digestion in both children and adults [17]. It can be used in various methods like ingestion, inhalation, or topical application. In the present study, all the children had taken EO as oral ingestion, which is a common mode of exposure. Only two children used EO as oral drops and topical application. Common cold was the most common reason to use the EO as a remedy in this present study.

The proconvulsant effect of EO can occur with accidental overdose, unintentional intake, or sometimes with therapeutic dosages. Most EO preparations are available OTC and in variable concentrations, hence it is difficult to decide the therapeutic dosage of EO. In this study, all the children used one liquid preparation which is available commonly in this location. One case series of EOIS in children was reported in the literature with this same EO preparation [9]. The neurological manifestations of EO exposure are broad-spectrum, ranging from mild confusion to life-threatening status epilepticus. The seizurogenic effect of EO is due to 1, 8-cineole, which also known as eucalyptol. Eucalyptol comprises at least 90% of the total contents of EO. The exact underlying mechanism of epileptogenesis is still not well reported. Studies have shown that epileptiform activity is induced by blockade of K+ channels and upregulation of Ca2+ inward currents [18-21]. The toxic effects of these compounds are more pronounced in children due to immature brains. Some of them have dose-related effects and some are idiosyncratic responses. When multiple compounds are present in a preparation, the complex interplay of all ingredients can cause toxic effects [22]. 

There are several case series and reports of EOIS in the literature worldwide. A study of 10 cases (five adults and five children) of EOIS was reported from South India [5]. Recently one more case series was reported with 15 children from South India with EOIS [9]. Our results are almost similar to these two studies. Mathew et al. reported EOIS due to inhalation of EO [5]. Flaman et al. reported EO ingestion causing seizures in children from 11 months to three years of age [13]. The onset-time of seizure ranged from 10 to 120 minutes after EO ingestion. Two cases of status epilepticus have been reported in six- and three-year-old boys after inges­tion of 5 and 10 mL of EO, respectively [14]. Seizures devel­oped in 3.5 hours after dermal application of EO for head lice removal have been reported [15]. Recently we published an article regarding three children with EOIS due to ingestion of EO for various reasons [23].

The Mathew et al. study reported that neuroimaging was normal in all the patients. In the present study, 10 children underwent neuroimaging, which was normal in all. The study by Ramesh et al. also reported similar neuroimaging findings. Mathew et al. reported EEG findings that are almost similar to our study. In our study, AEDs were used for two weeks, also like other studies. In this study, 10 children were hospitalized for evaluation. No children required intensive care management. During the follow-up period, none of the children had a recurrence of seizures which was similar to other studies [5]. All the parents were advised to avoid EO and EO-containing remedies.

Due to limited literature, less awareness about the proconvulsant properties of EO among health care professionals and caregivers are the major problems in the diagnosis of EOIS. That's why while approaching a child with the first episode of seizures or breakthrough seizures, the clinician needs to enquire about EO exposure. So many times, these seizures may be falsely labeled as idio­pathic seizures, and the patient could be advised for the long-term antiepileptic drugs unnecessarily.

To the best of our knowledge, this is the largest cohort of children to date, highlighting the seizurogenic potential of EO. This study had several limitations. First, this was a single-center study. Second, our small sample size could not represent all the pediatric population. Hence, the relationship between the EO and seizures needs to be further explored in large-scale prospective studies.

## Conclusions

In conclusion, EOIS is an underrecognized and rare entity of seizures in children. EOIS can occur as first seizures or as breakthrough seizures in preexisting epilepsy patients. Despite the previous case reports in the literature quoting the seizurogenic potential of EO, this awareness is lacking in both clinicians and parents. We also recommend clinicians inquire about the exposure to EO while approaching a child with seizures, which can prevent unnecessary investigations and long-term antiepileptic drug therapy.
